# Overcoming exhaustion: Building a conceptual foundation for nursing research

**DOI:** 10.1016/j.ijnss.2025.10.002

**Published:** 2025-10-15

**Authors:** Bridget Webb, Suzy Walter

**Affiliations:** a School of Nursing, West Virginia University, Morgantown, USA; b School of Nursing, Stevenson University, Owings Mills, USA

**Keywords:** Burnout, Concept building, Nurse, Overcoming exhaustion, Stress

## Abstract

**Objectives:**

This study aimed to establish the concept of overcoming exhaustion, providing a reference basis for nursing management and conducting related nursing research.

**Methods:**

Liehr and Smith’s three-phase, nine-step concept-building process was used to create the concept of overcoming exhaustion. The nine steps were as follows: 1) write a practice story; 2) name the emerging concept; 3) select a theoretical lens; 4) link concept to literature; 5) gather a concept story; 6) identify final core qualities; 7) formulate concept definition; 8) create a concept model; and 9) specify the concept building synthesis.

**Results:**

The concept of overcoming exhaustion was identified based on the elements of a practice story, the life experiences of nurses who struggle with the demands of caring for their patients, their families, and themselves. The theory of self-transcendence was then recognized as the theoretical lens from which to ground the concept. The core qualities, despair and moments of calmness, were derived from the literature and confirmed through a concept story. A definition of the concept integrating the core qualities was formed: overcoming exhaustion involves realizing moments of calmness amidst despair. A model was created to demonstrate the relationship between core qualities, despair, and moments of calmness.

**Conclusions:**

The concept of overcoming exhaustion was developed and, through the concept-building process, was defined as realizing moments of calmness amidst despair. By identifying the complexities of overcoming exhaustion, this work lays the foundation for a future research program to develop understanding and interventions that support nurse well-being in the context of ongoing personal and professional demands.

## What is known?

• Exhaustion is commonly associated with prolonged periods of stress and excessive physical exertion.
• Exhaustion is often discussed in the context of burnout and fatigue, but a unified concept of exhaustion is lacking.

## What is new?

• A three-phase, nine-step process defined a new concept of overcoming exhaustion following the identification of core qualities, including moments of calmness and despair.
• Information gained through each step of concept building can be used to understand better the experience of overcoming exhaustion.

## 1 Introduction

Nursing is experiencing a critical workforce crisis, with more nurses leaving the bedside than ever. Among the top reasons for this exodus is the deterioration of the work environment, which has led to an alarming rise in burnout [1]. According to a national survey [2], burnout was cited as the third leading cause for nurses leaving direct patient care in 2022, rising from fifth place in 2018. Burnout is characterized by Maslach and Jackson [3] as a sustained reaction to chronic job-related stressors. It includes three main dimensions: 1) exhaustion, 2) cynicism and detachment, and 3) lack of accomplishment. Currently, upwards of 90 % of nurses experience high levels of burnout [1]. Burnout can have a profoundly negative impact on nurses, leading to incivility, decreased job satisfaction, lower self-esteem, and a lack of motivation, which in turn further strains healthcare systems [4].

Exhaustion, particularly emotional exhaustion, is a foundational element of burnout. It extends beyond occasional tiredness, including persistent physical fatigue and psychological depletion [5]. Nurses are especially vulnerable to exhaustion due to the high demands, emotional labor, and intensity of clinical environments [6]. Emotional and physical fatigue are not only harmful to the well-being of nurses but also compromise the quality and safety of patient care. Research shows that emotional exhaustion is linked to an increase in adverse patient outcomes, including increased mortality rates, hospitalizations, and emergency department visits [6]. Emotional exhaustion and fatigue have also been identified as significant predictors of compromised patient safety [7]. Current research estimates up to 44 % of nurses experience emotional exhaustion, a condition associated with increased absenteeism, including the use of sick leave and mental health days [8]. These trends underscore the urgent need to address exhaustion as a distinct and significant symptom of burnout.

Despite its prevalence and consequences, exhaustion remains inconsistently defined and underconceptualized in the literature, which limits the development of targeted interventions to address this issue [6,7]. The potential negative consequences for patients and nurses underscore the importance of establishing a concept to overcome exhaustion. Clarifying this concept can guide future research, inform policy, and support the creation of evidence-based strategies to promote nurse wellbeing, retain bedside nurses, and ultimately improve patient outcomes. Nursing scholars can utilize concept building to conceptualize ideas derived from practice, drawing on both scientific and experiential evidence [9]. This paper aimed to guide the reader in understanding the concept of overcoming exhaustion, thereby enhancing comprehension of the life experiences of nurses who struggle with the demands of caring for their patients, their families, and themselves.

## 2 Methods

### 2.1 The method of establishing a concept

In this study, Liehr and Smith’s three-phase, nine-step concept-building process was employed to develop the concept of overcoming exhaustion [9]. The nine steps were as follows: 1) write a practice story; 2) name the emerging concept; 3) select a theoretical lens; 4) link concept to literature; 5) gather a concept story; 6) identify final core qualities; 7) formulate concept definition; 8) create a concept model; and 9) specify the concept building synthesis. The first phase of the process is grounding, which is extracting a concept rooted in nursing practice and includes the first three steps. From the nurse’s perspective, unearthing a concept from the ground up solidifies that concept in nursing, in caring in the human health experience [10]. Nursing practice and nurse-patient relationships are not only where knowledge is applied, but also where it is created [11]. This directly links nursing practice with the origin of knowledge. The value of the concept emerging from nursing practice lies in the connections between practice, concept, and theory, rooting the concept in nursing. The next phase is substantiating, and includes steps four, five, and six. This phase focuses on validating the concept with theoretical and empirical knowledge by gathering literature and information to support the emerging concept. The final phase of the concept-building process is resolution. This phase encompasses the last three steps of the process, finalizing the concept and core qualities. These steps provide direction for further research on the concept.

### 2.2 Ethical considerations

This concept-building process used a narrative storytelling approach in which the nurse recalls a critical incident from their practice that ties together a research interest and a passion. The narrative did not involve human subjects, so it fell outside the review of Institutional Review Board (IRB) oversight. However, the participant engaged in the concept story gathering was made aware of using the practice story for concept development. The participant was informed that the topic of the concept story was potentially exhausting, was given the option not to participate, and was assured of anonymity. Any recordings were deleted after the content was gathered. The practice story and the reconstructed narrative aimed to cultivate an empathetic understanding of human experience, fostering an appreciation for their unique perspectives. Furthermore, using the nine-phase process ensured the clinical nurse maintained an objective stance throughout the process.

## 3 Results

### 3.1 Phase 1: grounding

#### 3.1.1 Step 1: write a practice story

The first step in concept development is writing a practice story based on caring in the human health experience. Caring in the human health experience occurs in all areas of nursing practice (i.e., critical care, home health), allowing for the development of a concept that encompasses diverse nursing settings and experiences. Once written, peer input helped to clarify any confusing elements of the story [9].

The practice story was critiqued and analyzed with the help of colleagues. Input from colleagues with varied nursing perspectives allows discussion of any confusing elements of the practice story that need clarification [9]. Colleague input continues during Step 2 and involves naming the emerging concept, based on the practice story. Thus, collegial input begins with collaboration in refining the practice story and contributing to the identification of the concept. The practice story followed an ICU nurse preparing a patient for organ donation following a diagnosis of brain death due to cardiac arrest. The nurse must comfort the patient’s young daughter while still caring for her other patients, having slept little the night before. Demands at home, including caring for her newborn baby, and demands at work left the nurse exhausted. Going home, hugging her husband, and snuggling with her baby gave her some reprieve from that day.

#### 3.1.2 Step 2: name the emerging concept

The emerging concept was based on the experience of a nurse caring for critically ill patients and their family members, as well as for her own family. Based upon the elements of the practice story, the emerging concept began to develop. It was expressed as overcoming (hugging her husband, snuggling/kissing her baby), as well as exhaustion (waking up tired, unable to do it all, having a tough day), despite the demands of work and home. Therefore, the concept of overcoming exhaustion was identified.

#### 3.1.3 Step 3: select a theoretical lens

The next step is to select a theoretical lens, which serves as a guide for viewing the concept. Logical connections can be made from theory to the concept, establishing the concept within the discipline of nursing across various nursing backgrounds [9]. The philosophical perspective of intermodernism provided a guiding framework for the development of the concept. Intermodernism is described by Reed [12] as placing value on nursing practice, the community of scholars, and nursing theory. Nursing knowledge is developed from nursing practice, as highlighted by concept building. The perspective of intermodernism designates professional nursing practice as the foundation for creating and discovering knowledge and theory [12]. Integrating nursing practice into concept building helps solidify the notion that nursing practice needs to be central to knowledge development, theory, and innovation [12].

Intermodernism is woven into concept building through a theoretical lens, grounding nursing practice in theory. The theory of self-transcendence is the theoretical lens through which overcoming exhaustion can be viewed [13]. The central concepts in the theory of self-transcendence are self-transcendence, well-being, and vulnerability. Self-transcendence is the ability to go beyond one’s limitations and develop boundaries. Well-being is the idea of overall wellness and vitality, according to one’s beliefs. Vulnerability is the awareness of one’s own frailty in life. This theory supports the concept of overcoming exhaustion by describing that humans are integrated in their environment in all dimensions of reality (demands of work and home). Self-transcendence (coming home and being with family) is developmentally necessary for human growth and well-being [13]. Overcoming exhaustion is about expanding boundaries to find a moment of calmness (well-being, self-transcendence) amid despair (vulnerability).

### 3.2 Phase 2: substantiating

#### 3.2.1 Step 4: link concept to literature

A literature review was conducted to assess the current knowledge on the emerging concept and to identify its core qualities. Literature was searched using population-based, theoretical, and semantic approaches, enabling a comprehensive understanding of the emerging concept. To align with the central themes of this concept building, inclusion criteria for the literature review required that selected articles explicitly address at least one of the following key search terms: self-transcendence, exhaustion, burnout, emotional exhaustion, fatigue, despair, or calm. These terms were selected based on their relevance to the theoretical framework and their ability to capture both the physiological and psychological dimensions of exhaustion. Articles that did not meaningfully engage with these concepts were excluded. Databases searched were PubMed, CINAHL, Scopus, and PsycINFO. This search yielded 11 articles used for defining core quality. A literature matrix was used during the search to assist in identifying key elements and to categorize articles into population-based, theoretical, or semantic categories (Appendix A).

Identifying core qualities is based on recurring descriptors in the literature that represent various nursing disciplines (e.g., cardiac, ethics, critical care). Recurrent descriptors for moments of calmness included moments of mindfulness, purpose, peace, resilience, and growth [[14], [15], [16], [17]]. Recurrent descriptors of *despair* included feelings of emptiness, hopelessness, helplessness, as well as physical symptoms, including fatigue, pain, mood swings, and insomnia [[18], [19], [20]]. For theoretical support, self-transcendence has been shown to enhance the positive meaning derived from patient care experiences and overall well-being [15,21,22].

#### 3.2.2 Step 5: gather a concept story

Using the story-gathering approach [9], a concept story was developed, consisting of a beginning, middle, and end. The following is an excerpt from a concept story by a 40-year-old female nurse: She described her experience as pushing herself to the point of losing focus, becoming forgetful, redoing things, and feeling sick to her stomach from being so tired (despair). Meeting small goals helped her to get through the day, such as accomplishing one task at a time (moments of calmness). She felt a lot of pressure and exhaustion daily from overwhelming tasks and the never-ending work of being a nurse, working double shifts, and being a mom at home (despair). She enjoyed the weather, being in nature, spending time with family, and the smaller moments in life (moments of calmness). She described sitting on a balcony, listening to natural sounds, and appreciating the beauty and peace in that moment (moment of calmness).

The above concept story offered experiential evidence to support the core qualities of despair and moments of calmness. No other core qualities were drawn from the concept story.

#### 3.2.3 Step 6: identify final core qualities

The empirical evidence obtained from the literature and the concept story provided support for the core qualities, which were defined: 1) moments of calmness are peace, enjoyment, and growth [23,24]; 2) despair is a sense of hopelessness, unresolved grief, emptiness, fatigue, pain, and insomnia [19,20].

### 3.3 Phase 3: Resolving

#### 3.3.1 Step 7: formulate concept definition

The concept definition consists of the concept’s name followed by a careful arrangement of the core qualities [9]: overcoming exhaustion involves realizing moments of calmness in the midst of despair.

#### 3.3.2 Step 8: create a concept model

**Fig. 1:**
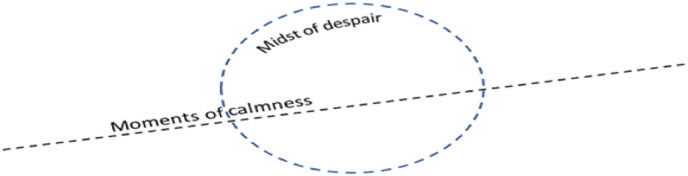
Conceptual model of overcoming exhaustion.

A model was created to visually represent the relationships between the core qualities. This model illustrates moments of calmness in a line that transitions through despair, conveying the concept of overcoming exhaustion. The lines and circles in the model are semi-porous and non-directional, allowing for the free movement of moments of calmness amidst despair (Fig. 1). The model highlights the selective permeability of the core qualities, allowing some moments of calmness to pass through while others are not, depending on the context. There is no predefined center of the model, highlighting the potential for despair and calmness to be equally active or not, depending on context.

#### 3.3.3 Step 9: specify the concept building synthesis

The final step in the concept-building process is to create a concept-building synthesis. The concept building synthesis is a three-sentence paragraph that succinctly describes the concept building process and provides guidance for research [9]. The synthesis is as follows: individuals immersed in demanding home and work environments can find ways to persevere on a daily basis. Overcoming exhaustion involves finding moments of calmness amidst despair. A qualitative study is proposed to gather the stories of nurses who continue to work despite the demands of work and home. A phenomenological approach could be employed to gain a deeper understanding of the unique lived experiences of nurses who struggle to meet the needs of their patients, families, and themselves [25].

## 4 Discussion

Overcoming exhaustion is a unique concept that acknowledges the importance of both the individual and the workplace environment in fostering moments of calmness. Concepts such as resilience focus primarily on the individual and their own use of coping strategies, but do not consider the role of the workplace environment in providing support [26].

Applying the concept of overcoming exhaustion in a healthcare setting will require individual and organizational interventions. Emotional exhaustion, fatigue, and burnout are now recognized as personal and organizational phenomena [27]. Individual interventions, such as mindfulness meditation, have the potential to decrease exhaustion and burnout, leading to moments of calmness [28]. Nurses working in hospitals and nursing homes are potentially at higher risk for emotional exhaustion due to insufficient teamwork [8]. Organizations can mitigate this by implementing strategies that reduce excessive workloads and help nurses manage emotional demands [8]. Organizational interventions should also include implementing an organizational-wide model, such as an evidence-based practice model like the Magnet model, that has been shown to have the potential to decrease burnout and improve patient outcomes by creating a culture of wellness [29,30]. Organizations can promote the importance of addressing burnout early during the training of new graduate nurses. For example, nurse residency programs should provide education about burnout and promote evidence-based interventions.

Both individual nurses and organizations can also address moments of calmness. Organizations can provide nurses with time and space to experience moments of calmness and emotional support. Creating cultural norms surrounding a team-centered atmosphere can provide moments of calmness for nurses. Team-centered culture can be protective against burnout, even showing evidence of providing resilience to providers [31]. Future interventions that strengthen team dynamics, clarify roles, and enhance care coordination could serve as key strategies for healthcare systems to reduce burnout among nurses and physicians [31].

The concept-building process was a challenging yet meaningful academic exercise. It required synthesis of diverse literature, critical thinking, and iterative reflection. Core qualities were identified, clarified, and integrated throughout the process to define the concept. Multiple rounds of analysis and refinement were necessary to describe the concept’s unique attributes and ensure it was well-defined, meaningful, and connected to the present theory. This work transformed a broad and abstract idea into a defined concept with practical and theoretical relevance.

Concept building can guide nurses into beginning a research program. Future research can focus on the concept of overcoming exhaustion, specifically on the core qualities, and how individuals, environments, and organizations can serve as catalysts to create moments of calmness and mitigate despair. A descriptive mixed-methods study investigating ways to overcome exhaustion in nurses would help healthcare providers and systems better support and understand the needs of their employees and coworkers. A qualitative investigation into what provides moments of calmness could provide insight into a better understanding of the concept. This study would include both quantitative data and qualitative interviews, allowing nurses to express their thoughts and feelings, and help expand the concept of overcoming exhaustion. Recruitment strategies may consist of traditional approaches, such as distributing flyers, sending emails, and displaying on-site advertisements. Funding could be secured for incentives, such as gift cards, for the participants. Findings could inform the development of tailored interventions, policies, and wellness programs.

## 5 Conclusions

The concept of overcoming exhaustion was developed and, through the concept-building process, was defined as realizing moments of calmness amidst despair. Core qualities of the concept were identified, including moments of calmness and despair, through a thorough literature review. The concept was theoretically anchored in the established framework of the theory of self-transcendence. The significance of building this concept lies in its contribution to understanding a deeply relevant phenomenon in nursing practice. This structured approach provides nursing scholars with the opportunity to take ideas from practice and develop a concept related to an area of research interest. By identifying the complexities of overcoming exhaustion, this work lays the foundation for a future research program to develop understanding and interventions that support nurse well-being in the context of ongoing personal and professional demands.

## Data availability statement

Data sharing is not applicable to this article as no datasets were generated or analyzed during the current study.

## CRediT authorship contribution statement

**Bridget Webb:** Conceptualization, Methodology, Writing - original draft, Writing - review & editing. **Suzy Walter:** Conceptualization, Methodology, Writing - review & editing.

## Declaration of competing interest

There are no conflicts of interest to be noted for this manuscript.

## References

[1] Galanis P., Moisoglou I., Katsiroumpa A., Vraka I., Siskou O., Konstantakopoulou O. (2023). Increased job burnout and reduced job satisfaction for nurses compared to other healthcare workers after the COVID-19 pandemic. Nurs Rep.

[2] U.S. Census Bureau (2024). 2022 National sample survey of registered nurses methodology report. https://data.hrsa.gov/topics/health-workforce/nchwa/nursing-workforce-survey-data.

[3] Maslach C., Jackson S.E. (1981). The measurement of experienced burnout. J Organ Behav.

[4] Kelly L.A., Gee P.M., Butler R.J. (2021). Impact of nurse burnout on organizational and position turnover. Nurs Outlook.

[5] Dall'Ora C., Ball J., Reinius M., Griffiths P. (2020). Burnout in nursing: a theoretical review. Hum Resour Health.

[6] Beaulieu L., Seneviratne C., Nowell L. (2023). Change fatigue in nursing: an integrative review. J Adv Nurs.

[7] Al Ma’mari Q., Sharour L.A., Al Omari O. (2020). Fatigue, burnout, work environment, workload and perceived patient safety culture among critical care nurses. Br J Nurs.

[8] Petersen J., Wendsche J., Melzer M. (2023). Nurses' emotional exhaustion: prevalence, psychosocial risk factors and association to sick leave depending on care setting-a quantitative secondary analysis. J Adv Nurs.

[9] Liehr P.R., Smith M.J. (2024).

[10] Newman M.A., Sime A.M., Corcoran-Perry S.A. (1991). The focus of the discipline of nursing. ANS Adv Nurs Sci.

[11] Reed P.G. (2006). The practice turn in nursing epistemology. Nurs Sci Q.

[12] Reed P.G. (2019). Intermodernism: a philosophical perspective for development of scientific nursing theory. ANS Adv Nurs Sci.

[13] Reed P.G. (1991). Toward a nursing theory of self-transcendence: deductive reformulation using developmental theories. ANS Adv Nurs Sci.

[14] Lundgren O., Garvin P., Kristenson M., Jonasson L., Thylén I. (2018). A journey through chaos and calmness: experiences of mindfulness training in patients with depressive symptoms after a recent coronary event - a qualitative diary content analysis. BMC Psychol.

[15] Aydın M., Aydın Avci İ., Kulakaç Ö. (2022). Nurses as the leading fighters during the COVID-19 pandemic: self-Transcendence. Nurs Ethics.

[16] Hawthorne D.M., Barry C.D. (2021). Nurses' use of spiritual practices in caring for self during the pandemic. Holist Nurs Pract.

[17] Aycock N., Boyle D. (2009). Interventions to manage compassion fatigue in oncology nursing. Clin J Oncol Nurs.

[18] Connor M.J., Walton J.A. (2011). Demoralization and remoralization: a review of these constructs in the healthcare literature. Nurs Inq.

[19] Younas A., Rasheed S.P. (2018). Compassionate self-awareness: a hidden resource for nurses for developing a relationship with self and patients. Creat Nurs.

[20] Milutinović D., Golubović B., Brkić N., Prokeš B. (2012). Professional stress and health among critical care nurses in Serbia. Arh Hig Rada Toksikol.

[21] Palmer B., Quinn Griffin M.T., Reed P., Fitzpatrick J.J. (2010). Self-transcendence and work engagement in acute care staff registered nurses. Crit Care Nurs Q.

[22] Hwang H.L., Tu C.T., Chan H.S. (2019). Self-transcendence, caring and their associations with well-being. J Adv Nurs.

[23] Fiske E.A. (2019). Self-transcendence, well-being, and vulnerability in healthcare mission participants. Nurs Sci Q.

[24] Groves K.A., Adewumi A., Gerhardt C.A., Skeens M.A., Suttle M.L. (2022). Grief in critical care nurses after pediatric suffering and death. Ann Palliat Med.

[25] Van Manen M. (1997).

[26] Morse J.M., Kent-Marvick J., Barry L.A., Harvey J., Okang E.N., Rudd E.A. (2021). Developing the resilience framework for nursing and healthcare. Glob Qual Nurs Res.

[27] Jun J., Ojemeni M.M., Kalamani R., Tong J., Crecelius M.L. (2021). Relationship between nurse burnout, patient and organizational outcomes: systematic review. Int J Nurs Stud.

[28] Green A.A., Kinchen E.V. (2021). The effects of mindfulness meditation on stress and burnout in nurses. J Holist Nurs.

[29] Gonçalves I., Mendes D.A., Caldeira S., Jesus É., Nunes E. (2023). The primary nursing care model and inpatients' nursing-sensitive outcomes: a systematic review and narrative synthesis of quantitative studies. Int J Environ Res Publ Health.

[30] O'Hara S., Melnyk B.M., Hsieh A.P., Helsabeck N.P., Giuliano K.K., Vital C. (2025). Innovation, wellness, and EBP cultures are associated with less burnout, better mental health, and higher job satisfaction in nurses and the healthcare workforce. Worldviews Evid Based Nurs.

[31] Lu M.A., O'Toole J., Shneyderman M., Brockman S., Cumpsty-Fowler C., Dang D. (2023). “where you feel like a family instead of co-workers”: a mixed methods study on care teams and burnout. J Gen Intern Med.

