# Supplementary material for: Overcoming exhaustion: Building a conceptual foundation for nursing research
Source: Int J Nurs Sci. 2025 Oct 15;12(6):588–92. doi: 10.1016/j.ijnss.2025.10.002 (PMC12684752; doi:10.1016/j.ijnss.2025.10.002)
Supplement: Multimedia component 1 [file mmc1.docx]

**护士克服疲劳的概念构建研究**

Bridget Webb, Suzy Walter

**【摘要】**

**目的** 构建护士克服疲劳的概念，为护理管理及开展相关研究提供参考依据。

**方法** 采用利尔和史密斯提出的三阶段、九步骤的概念构建方法，包括：1）撰写实践故事；2）概念命名；3）选择理论视角；4）将概念与文献联系起来；5）收集概念故事；6）确定核心特质；7）明确概念定义；8）创建概念模型；9）形成综合内容。

**结果** 基于同时照顾患者、家人以及自身方面面临巨大压力护士的实际生活经历，确定了克服疲劳的概念。随后，选用自我超越理论作为理论框架，并从相关文献中提炼出克服疲劳的核心特质：绝望与平静时刻，进一步通过概念故事予以验证。最终形成整合性概念定义：克服疲劳是指在持续的职业与个人压力中，经历绝望的同时仍能寻获内心平静的动态过程。

**结论** 该研究成功构建了“克服疲劳”的概念，通过系统阐释该概念的复杂内涵，为未来开发相关干预措施、促进护士在持续压力背景下的身心健康奠定了理论基础。

**【关键词】**职业倦怠；概念构建；护士；克服疲劳；压力

**通信作者：**Bridget Webb, E-mail: [bwebb15@tnstate.edu](mailto:bwebb15@tnstate.edu)
