# Supplementary material for: Overcoming exhaustion: Building a conceptual foundation for nursing research
Source: Int J Nurs Sci. 2025 Oct 15;12(6):588–92. doi: 10.1016/j.ijnss.2025.10.002 (PMC12684752; doi:10.1016/j.ijnss.2025.10.002)
Supplement: Multimedia component 2 [file mmc2.docx]

Appendix A Literature matrix for core qualities.

| Author | Journal | Title | Core quality and definition | Article type |
| --- | --- | --- | --- | --- |
| Lundgren et al., 2018 [14] | BMC Psychology | A journey through chaos and calmness: Experiences of mindfulness training in patients with depressive symptoms after a recent coronary event - a qualitative diary content analysis | Moments of calmness: mindfulness, paying attention, on purpose, in the present moment, and as non-judgmentally as possible, intention, attention, and attitude, relation to an experience—a journey through calmness | Population-based |
| Aydin et al., 2022 [15] | Nursing Ethics | Nurses as the leading fighters during  the COVID-19 pandemic:  Self-transcendence | Despair: anxiety, low job performance, and satisfaction  Moments of Calmness: expand boundaries, grow spiritually, connect with others around  Self-transcendence played a role in nurses’ mental and physical well-being | Theoretical, Population-based |
| Hawthorne and Barry 2012 [16] | Holistic Nursing Practice | Nurses’ use of practices on caring for self during the pandemic | Despair: stressful life events, stressful work environments, social isolation, exhaustion, despondence, hopelessness, crisis.  Moments of calmness: spiritual practice | Population-based, semantic |
| Aycock and Boyle 2009 [17] | Clinical Journal of Nursing Oncology | Interventions to manage compassion  fatigue in oncology nursing | Despair: compassion fatigue, severe malaise  Moments of calmness: humor, responsible selfishness | Population-based, Semantic |
| Connor and Walton 2018 [18] | Nursing Inquiry | Demoralization and remoralization:  a review of these constructs in the  healthcare literature | Despair: poor morale, low self-esteem, social failure, hopelessness, helplessness, isolation, entrapment | Population-based |
| Younas and Rasheed 2012 [19] | Creative Health Care Management | Compassionate self-awareness:  A hidden resource for nurses for  developing a relationship with self  and patients | Despair: inability to provide compassion, feelings of emptiness, hopelessness, physical  tiredness, incompetence, frustration | Population-based |
| Milutinovic et al., 2012 [20] | Arh Hig Rada Toksiko | Professional stress and health among  critical care nurses in Serbia | Despair: physical manifestations such as headache, insomnia, fatigue, despair, lower back pain, frequent mood swings, excessive sweating, shortness of breath, chest pain, and palpitations | Population-based |
| Palmer et al., 2010 [21] | Critical Care Nursing Quarterly | Self-transcendence and work  engagement in acute care  staff registered nurses | The theory of self-transcendence was used as a foundation to increase work engagement, finding that nurses were more content and better able to help patients | Population-based, theoretical |
| Hwang et al., 2019 [22] | Journal of Advanced Nursing | Self‐transcendence, caring and their associations with  well‐being | Self-transcendence is both a personal resource and a continual struggle for well-being. Vulnerability is defined as a challenge in life. | Theoretical, Semantic |
| Fiske 2019 [23] | Nursing Science Quarterly | Self-Transcendence, well-being, and  vulnerability in healthcare mission  participants. | Through self-transcendence, people find meaning in life’s  struggle, expand their boundaries, and connect with themselves and their spiritual dimensions | Theoretical |
| Groves et al., 2022 [24] | Annals of Palliative Medicine | Grief in critical care nurses after pediatric suffering and death | Despair: unresolved grief, cumulative grief, poor self-esteem, depression, apathy, physical illness, suicidal thoughts  Moments of calmness: resilience, appreciating family, encompassing optimism, realism, self-efficacy, and humor | Population-based |
